# The Use of Vacuum Devices as Adjuvant Therapy before and after Penile Curvature Surgery in Patients Affected by La Peyronie’s Disease: Results from a Comparative Study

**DOI:** 10.3390/clinpract13050112

**Published:** 2023-10-17

**Authors:** Tommaso Cai, Marco Capece, Carlo Ceruti, Daniele Tiscione, Marco Puglisi, Paolo Verze, Paolo Gontero, Alessandro Palmieri

**Affiliations:** 1 Department of Urology, Santa Chiara Hospital, 38122 Trento, Italy; daniele.tiscione@apss.tn.it (D.T.); marco.puglisi@apss.tn.it (M.P.); 2 Institute of Clinical Medicine, University of Oslo, 0313 Oslo, Norway; 3 Department of Urology, University of Naples, Federico II, 80138 Naples, Italy; drmarcocapece@gmail.com (M.C.); info@alessandropalmieri.it (A.P.); 4 Department of Urology, University of Turin, 10124 Turin, Italy; carlo.ceruti@unito.it (C.C.); paolo.gontero@unito.it (P.G.); 5 Department of Urology, University of Salerno, 84084 Salerno, Italy; pverze@gmail.com

**Keywords:** Peyronie’s disease, vacuum erection devices, penile curvature, surgery, quality of life

## Abstract

Background: Peyronie’s disease (PD) represents a challenging urological disease, due to not optimal post-operative surgical outcomes. We aim to retrospectively evaluate if vacuum erection device (VED) treatment before penile curvature surgery is able to improve post-operative surgical outcomes. Methods: All enrolled patients were assigned to the following groups: (a) the treatment group: VED treatment (three times per week) starting 3 months before surgery and (three times per week) one month after surgery; and (b) the control group: VED treatment (three times per week) one month after surgery. Follow-up urologic visits were scheduled for 3 and 6 months after surgery, and the two groups were compared. Results: A total of 38 patients were enrolled (median age 67 years, 57–74, IQR): 20 in the treatment group and 18 in the control group. At the follow-up visits, the two groups were different in terms of IIEF-5 (26 vs. 24; *p* = 0.02), “yes” to SEP2 and 3 (85% vs. 55%; *p* < 0.001, 85% vs. 50%; *p* < 0.001, respectively), and PDQ (−16 vs. −11; *p* = 0.03). Complete correction of penile curvature was achieved in 36 patients (94.7%). In the treatment group, no hourglass deformity was reported, whereas one patient reported a mild hourglass deformity in the control group. In the treatment group, we obtained a longer total penile length (median +1.5 cm). The overall satisfaction rate was 98% in the treatment group and 96% in the control group. Conclusions: The VED treatment before penile curvature surgery in patients affected by PD was able to improve surgical outcomes.

## 1. Introduction

Peyronie’s disease (PD) represents a challenging andrological disease with an important impact on patients’ quality of life [1]. The social burden of the disease is high due to its prevalence among sexually active males, ranging between 0.4 and 20%, and its chronic evolution, with a high frequency of recurrence and non-optimal efficacy in the current therapeutic strategies [2,3,4]. Moreover, its natural history is not fully understood [5]. Patient satisfaction and surgery outcomes are not always optimal, and several medical and surgical treatment options are purposed and introduced in everyday clinical practice [4]. In the chronic stable phase of the disease, surgery remains the recommended approach [6]. The most common surgical technique for the management of PD is plaque incision and grafting, with a reported success rate between 87.5% and 100% [4]. The use of vacuum erection devices (VEDs) is recommended due to their capabilities to obtain dilations of cavernous sinuses, decreased retrograde venous blood flows, and increased arterial inflows [7]. International guidelines suggest using VEDs as part of a multimodal approach, highlighting that combination therapy is more effective when compared to monotherapy [8]. Here, we focus our attention on the roles of VEDs as adjuvant therapies before surgeries in patients with the chronic, stable phase of the disease. Even under the same pre-operative conditions, such as sizes and locations of penile plaques, degrees of curvature, complex deformities (hinge or hourglass), penile lengths, and the presences or absences of erectile dysfunction, the surgical outcomes and patient’s satisfaction are not fully associated with the surgical technique and the surgeon’s skill. Several authors highlighted the role of post-operative penile rehabilitation to improve surgical outcomes by using VEDs or penile traction devices [8,9,10]. Moreover, the use of VEDs has been recommended for the management of post-operative complications such as penile shortening, penile deformities, or residual curvatures [10]. On the basis of these considerations, we aim to evaluate if VED treatment before penile curvature surgery is able to improve post-operative surgical outcomes.

## 2. Materials and Methods

### 2.1. Study Design and Patient’s Population

In this study, we included all consecutive adult patients with Peyronie’s disease who were treated by plaque incision and bovine pericardium grafting between January 2020 and September 2022 in an Italian referral center. All patients underwent 3 months of VED treatment (3 times per week) before penile curvature surgery and then VED treatment (3 times per week) starting one month after surgery. At 6 months after the surgical procedure, all patients were scheduled for a follow-up visit with dedicated questionnaires. The results of this group of patients were compared with a cohort of patients who were treated by plaque incision and bovine pericardium grafting between January 2017 and December 2019 at the same institution and treated with VED treatment only after surgical treatment. The two groups were compared in terms of patient satisfaction and surgical outcome. In January 2020, we introduced another protocol with preoperative VED treatment in all patients affected by La Peyronie’s disease and candidates for surgery, due to the recent evidence that demonstrated the role of VEDs in smooth muscle presentation and fibrosis reduction by decreased TGF-b1 expression [11]. In this sense, the use of VEDs could have a greater effect on erectile function maintenance by decreases in TGF-b1 and cell apoptosis [11].

**Figure 1:**
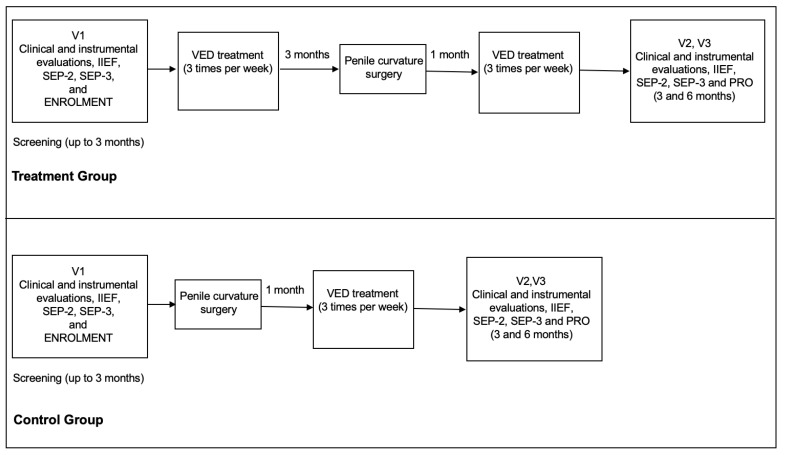
The figure shows the study schedule. IIEF-5: International Index of Erectile Function Questionnaire; SEP: the Sexual Encounter Profile; PRO: Patient Reported Ouitcome.

Figure 1 shows the study schedule.

### 2.2. Inclusion and Exclusion Criteria

Patients were considered for this study if they matched the following characteristics: had a chronic stable phase of the disease, were refractory to conservative management, and had a severe curvature (>60°) or penile complex deformity (hourglass or hinge) without any evidence of erectile dysfunction (International Index of Erectile Function Questionnaire-5 more than 22). We excluded all patients with PD in the active phase, erectile dysfunction, congenital penile curvature, or significant medical conditions.

### 2.3. Preoperative Assessment and VED Adjuvant Treatment

All patients, including both groups, underwent preoperative evaluation with detailing their medical history, physical examinations, the International Index of Erectile Function Questionnaire (IIEF-5), the Peyronie’s Disease Questionnaire (PDQ), a measurement of penile length during relaxation and erection, and penile Doppler ultrasound during prostaglandin-induced erection, in line with the recommendations of the international guidelines [8]. Penile Doppler ultrasound was performed by intra-cavernous injection of 10 mcg alprostadil to assess the angle and direction of penile curvature. After the inclusion in the study, all patients were counselled about the use of VEDs, according to the group. All patients underwent vacuum erection therapy by using the same device from MEDIS, Medical Service—Rozzano, Milan, Italy.

### 2.4. Surgical Treatment and Post-Operative Management

All surgical procedures were performed by the same urologists (T.C. and D.T.) by using the same surgical technique in both treatment periods. In brief, after penile degloving, the Buck’s fascia was carefully divided and the dorsal neurovascular bundle mobilized. Erection was intraoperatively obtained by injecting 0.9% NaCl into the corpus cavernosum. A double “Y” shape incision was performed at the maximum curvature. The wound edges were expanded as much as possible, and the distance between the edges of the defect were measured by a ruler. Bovine pericardium graft (Bovine pericardium tissue—Baxter, Italy) was placed on the resulting defect and was sutured with continuous running sutures with the MonoPlus^®^ No. 4 (B. Braun, Milan, Italy). No oversizing of the graft was performed. Artificial erection was induced in order to check the curvature correction and the presence of residual penile deformities. The Buck’s fascia and the skin were sutured, and a compressive penile bandage was performed after Foley catheter placement. The catheter and penile bandage were removed 24 h after surgery, and the patient was immediately discharged. All patients underwent phosphodiesterase type-5 inhibitors therapy (tadalafil 5 mg 1 tablet per day) for 3 months starting immediately after surgery. Moreover, starting 30 days after surgery, all patients were asked to continue VED therapy (3 times per week). All patients were asked to avoid sexual intercourse for 6 weeks. Follow-up visits were scheduled at 30 days, 3 months, and 6 months after surgery. A 30 day visit was performed to test short-term surgical complications, to check the adherence to phosphodiesterase type-5 inhibitors therapy, and to counsel the patients for the future therapy and follow-up visits. At 3 and 6 months follow-up visits, all patients underwent urological visits, penile lengths, and evaluations of eventual residual curvature and penile deformities by using self-made photography with an erected penis. Moreover, all patients underwent IIEF-5 questionnaires and Patient Reported Outcomes (PROs).

### 2.5. Outcome Measurements

The primary outcome was to evaluate superiority of neo-adjuvant therapy with VEDs in patients’ candidates to penile curvature surgeries when compared with penile curvature surgery alone, in terms erectile function domain score (International Index of Erectile Function, PDQ, SEP-2, and SEP-3) and patients satisfaction (PROs). Moreover, the penile length change was considered a secondary outcome measure.

### 2.6. Questionnaires

Efficacy measures were the International Index of Erectile Function and the Sexual Encounter Profile (SEP) questionnaires [12,13,14], collected for each patient at the baseline and at the end of each treatment phase and at the follow-up evaluation. The Peyronie’s Disease questionnaire (PDQ) was also used [15].

### 2.7. Statistical and Ethical Considerations

Demographic and baseline characteristics were presented as numbers and percentages. Numeric variables were shown as mean ± standard deviation when normally distributed, and median and interquartile range in cases of not normal distribution. The McNemar’s test was used to compare categorical variables. The Friedman test was used in case of not normally distributed variables. The changes between the two groups in the EF domain score and EF questions 3 and 4 scores were analyzed using a covariance (analysis of covariance) model, with treatment and study site as the main factors, and the baseline EF domain score as the covariate. Responses to the SEP questions were analyzed using a one-way analysis of variance with treatment as a factor. Two-tailed *p* values less than 0.05 were considered statistically significant. All statistical analyses were performed using SPSS 22 for the Apple Mac (SPSS, Inc., Chicago, IL, USA). However, all anamnestic, clinical, and laboratory data containing sensitive information about patients were de-identified to ensure the analysis of anonymous data only. The de-identification process was performed by non-medical staff by means of dedicated software [16]. The study was conducted in line with the Good Clinical Practice guidelines and the ethical principles laid down in the latest version of the Declaration of Helsinki.

## 3. Results

Twenty patients were enrolled from January 2020 to September 2022 (treatment group) (median age 67 years, 57–74, Interquartile range (IQR)) and compared with 18 patients enrolled from January 2017 to December 2019 (control group) (median age 66 years, 54–75, IQR).

### 3.1. Clinical Characteristics of the Study Participants at the Enrolment

Dorsal curvature was the most frequent site of curvature (65%). The median curvature at preoperative evaluation was 65° (ranging from 60 to 80°). All patients had stable-phase PD, at least for the preceding 12 months. Each patient received previous medical/conservative treatment without any clinical success. No patient received VED treatment alone or in association with other therapies before enrolment. The IIEF-15 median score was 23, ranging from 22 to 24. The median erect penile length at preoperative evaluation was 114 mm (110–140, IQR).

Table 1 shows all clinical and anamnestic characteristics of all patients at enrolment. 

### 3.2. Intra-Operative and Peri-Operative Findings

No clinically significant differences were reported between the VED treatment starting and the surgical time in both groups. The median operative time was 105 min (84–132, IQR). Intraoperative complications were very low, in only one case a lesion of neurovascular bundle occurred. The lesion was repaired with VICRYL^®^ No. 4 (ETHICON SPA Pomezia, Rome).

Table 2 shows all intra-operative and peri-operative findings. 

Complete curvature correction (residual curvature < 10°) was achieved in 36 patients (94.7%). The median penile length obtained at the end of the surgical procedure was 116 mm (112–140, IQR) in the treatment group while 114 (110–140, IQR) in the control group.

### 3.3. Follow-Up Results

All patients regularly used the VED. A statistically significant improvement of IIEF-5 (26 vs. 24; *p* = 0.02), “yes” to SEP2 and 3 (85% vs. 55%; *p* < 0.001, 85% vs. 50%; *p* < 0.001, respectively), was reported between the two groups. Moreover, the PDQ overall score significantly improved between the two groups (−16 vs. −11; *p* = 0.03). Thirty-five patients maintained a complete correction of penile curvature (zero degrees) at 6 months (92.1%). In the treatment group, no hourglass deformities were reported. On the other hand, in the control group, one patient reported a mild hourglass deformity at 6 months of follow-up. Finally, in the treatment group, we obtained a longer total penile length (median + 1.5 cm) in comparison with the control group. The overall satisfaction rate was 98% in the treatment group and 96% in the control group, on the basis of the Patient Reported Outcomes. Twenty-five patients (65.7%) had normal penile sensitivity, whereas 13 (34.3%) reported mild hypesthesia. Fifteen patients (39.4%) continued to use on-demand tadalafil 20 mg for 6 months from surgery.

Table 3 shows all clinical and questionnaires results at 6 months follow-up visit. 

## 4. Discussion

The use of xenografts for the surgical management of PD has recently become popular and commonly used [17], even if this approach is associated with some possible risks, such as penile shortening, de novo erectile dysfunction, glans hypoaesthesia or anaesthesia, and residual or recurrent curvature [10,18]. In this sense, the use of vacuum devices could be useful for reducing the risk of complications and to improve the patients’ outcomes and satisfaction.

### 4.1. Main Findings

Here, we demonstrated that VED treatment before penile curvature surgery in patients affected by PD was able to improve post-operative surgery in terms of patient satisfaction and surgical outcome. To the best of our knowledge, this is the first study evaluating the role of VED treatment as adjuvant therapy to improve surgical outcomes in PD patients.

### 4.2. Results in the Context of Current Knowledge

The authors of the ESSM Position Statement on Surgical Treatment of Peyronie’s Disease highlighted that the aim of surgery is to improve penile deformities whilst minimizing any adverse outcomes [10]. In order to reduce the risk of complications and improve the outcome of surgery, penile traction therapy and VED techniques have been proposed [19]. VED therapy is able to improve dilations of cavernous sinuses; retrograde venous blood flow; increased arterial inflow, resulting in the decreasing of hypoxia-inducible factor-1a, transforming growth factor (TGF)-b1, collagenase, and apoptosis; and increases in endothelial nitric oxide synthase and a-smooth muscle actin [19,20]. Only one study with a limited number of enrolled patients has been published on the adjunctive use of a VED after incision and grafting surgery for PD [21]. In this study, the patients were encouraged to use a VED 30 min daily for 6 months, starting 1 month after surgery [21]. The authors demonstrated that patients treated with VED gained 2 inches in penile length compared with 1 inch in the man not using VED therapy. In this study, the authors evaluated the role of VED therapy after surgery as an adjuvant procedure. In our experience, the use of VED beginning 3 months before the surgery was able to improve surgical outcome, probably by inducing the dilation of cavernous sinuses and increased arterial inflow. Another aspect to discuss is the role of VED in the management of penile curvature, regardless of surgery. In our experience, no clinically significant differences were reported between the VED treatment starting and the surgical time in both groups, demonstrating that patient satisfaction was related to the use of VED treatment before penile curvature surgery.

### 4.3. Strengths and Limitations of This Study

There were some limitations associated with this study. The nature of the study, the retrospective and non-randomized clinical trials, and a lack of long-term follow-up evaluation should be considered as limitations. The strengths of this study were the fact that the study was conducted in a high volume andrological center and the use of a standardized surgical approach to the PD.

## 5. Conclusions

In conclusion, VED treatment before penile curvature surgery in patients affected by PD could be a factor of improved postoperative outcomes in terms of patient satisfaction and surgical outcome. Further studies with randomized and controlled features are required in order to confirm our results. Moreover, future studies investigating the histologic and molecular changes in tunica albuginea and corpora cavernosa after VED could give informative data on the role of VED as adjuvant therapy to surgery in PD patients.

## Figures and Tables

**Table 1:** Clinical and anamnestic characteristics of all patients at the enrolment.

	Treatment	Control	*p*
No. of patients	20	18	
Age (years)			1
Median (IQR ^†^)	67 (57–74)	66 (54–75)	
Marital status			0.99
Married	18 (90)	17 (94.4)	
Single	2 (10)	1 (5.6)	
Duration of disease-time from onset (months)			0.89
Median (IQR ^†^)	13 (12–22)	14 (13–21)	
Penile curvature			0.94
Angle, degrees (IQR ^†^)	66 (60–80)	64 (60–80)	
Direction, no.			
dorsal	13 (65)	12 (66.6)	
lateral	4 (20)	5 (27.7)	
ventral	3 (15)	1 (5.7)	
Penile deformity			0.77
Hourglass	3 (15)	2 (11.1)	
Penile length (mm)			0.83
Median (IQR ^†^)	113 (110–140)	115 (110–140)	
Plaque size (mm)			0.94
Median (IQR^†^)	21 (15–31)	22 (16–35)	
IIEF-15 *			0.82
Median (IQR ^†^)	24 (22–24)	22 (22–24)	
PDQ °			0.75
Median (IQR ^†^)	28 (19–35)	28 (18–34)	
PDQ-Penile Pain	13 (1–23)	13 (1–23)	
PDQ-Symptom Bother	10 (5–16)	9 (4–16)	
PDQ-Psychological and Physical Symptoms	3 (0–20)	4 (0–20)	
SEP-2 §			0.65
Yes	11 (55)	9 (50)	
SEP-3 §			0.84
Yes	10 (50)	9 (50)	

IQR ^†^ = interquartile range; IIEF-15 * = International Index of Erectile Function; PDQ ° = Peyronie’s Disease Questionnaire; SEP § = Sexual Encounter Profile. Data in parentheses are percentages unless otherwise specified.

**Table 2:** Intra-operative and peri-operative findings.

	Treatment	Control	*p*
Operative time (min)			0.98
Median (IQR ^†^)	105 (84–132)	103 (84–128)	
Complete curvature correction			0.57
No.	19 (95)	17 (94.4)	
Penile length (mm)			0.41
Median (IQR ^†^)	116 (112–140)	114 (110–140)	
Complications			0.95
Hematoma	3 (15)	3 (16.6)	
Oedema	9 (45)	8 (40)	

IQR ^†^ = interquartile range. Data in parentheses are percentage unless otherwise specified.

**Table 3:** Clinical and questionnaires results at 12 months follow-up visit.

	Treatment	Control	*p*
VED ° use (regular)			1
No.	20 (100)	18 (100)	
Patient Reported Outcomes			0.02
Overall satisfaction	19 (95)	14 (77.7)	
Penile sensitivity			0.03
Normal	14 (70)	11 (61.1)	
IIEF-15 * pre-			0.82
Median (IQR)	24 (22–24)	22 (22–24)	
IIEF-15 * post-			0.02
Median (IQR)	26 (22–24)	24 (22–24)	
*p* = 0.03			
SEP-2 § pre-			0.65
Yes	11 (55)	9 (50)	
SEP-2 § post-			<0.001
Yes	17 (85)	12 (66.6)	
*p* < 0.001			
SEP-3 § pre-			0.84
Yes	11 (55)	9 (50)	
SEP-3 § post-			<0.001
Yes	17 (85)	13 (72.2)	
*p* < 0.001			
PDQ ° overall—pre-			0.75
Median (IQR)	28 (19–35)	28 (18–34)	
PDQ ° overall—post-			0.03
Median (IQR)	12 (8–20)	17 (16–28)	
*p* < 0.001			

VED ° = vacuum erection devices; IIEF-15 * = International Index of Erectile Function; SEP § = Sexual Encounter Profile; PDQ ° = Peyronie’s Disease Questionnaire. Data in parentheses are percentage unless otherwise specified.

## Data Availability

All clinical data are unavailable due to privacy or ethical restrictions by Italian bylaw.

## References

[1] Moisés da Silva G.V., Dávila F.J., Rosito T.E., Martins F.E. (2022). Global Perspective on the Management of Peyronie’s Disease. Front. Reprod. Health.

[2] Kadioglu A., Dincer M., Salabas E., Culha M.G., Akdere H., Cilesiz N.C. (2020). A Population-Based Study of Peyronie’s Disease in Turkey: Prevalence and Related Comorbidities. Sex. Med..

[3] Stuntz M., Perlaky A., des Vignes F., Kyriakides T., Glass D. (2016). The Prevalence of Peyronie’s Disease in the United States: A Population-Based Study. PLoS ONE.

[4] Ainayev Y., Zhanbyrbekuly U., Gaipov A., Suleiman M., Kadyrzhanuly K., Kissamedenov N., Zhaparov U., Akhmetov D., Khairli G. (2021). Surgical Reconstruction of Penile Curvature due to Peyronie’s Disease by Plaque Incision and Buccal Mucosa Graft. J. Sex. Med..

[5] Milenkovic U., Ilg M.M., Cellek S., Albersen M. (2019). Pathophysiology and Future Therapeutic Perspectives for Resolving Fibrosis in Peyronie’s Disease. Sex. Med. Rev..

[6] Chung E., Gillman M., Tuckey J., La Bianca S., Love C. (2020). A clinical pathway for the management of Peyronie’s disease: Integrating clinical guidelines from the International Society of Sexual Medicine, American Urological Association and European Urological Association. BJU Int..

[7] Broderick G.A., McGahan J.P., Stone A.R., White R.D. (1992). The hemodynamics of vacuum constriction erections: Assessment by color Doppler ultrasound. J. Urol..

[8] European Association of Urology Guidelines on Sexual and Reproductive Health. https://d56bochluxqnz.cloudfront.net/documents/full-guideline/EAU-Guidelines-on-Sexual-and-Reproductive-Health-2022_2022-03-29-084141_megw.pdf.

[9] Rybak J., Papagiannopoulos D., Levine L. (2012). A retrospective comparative study of traction therapy vs. no traction following tunica albuginea plication or partial excision and grafting for Peyronie’s disease: Measured lengths and patient perceptions. J. Sex. Med..

[10] Osmonov D., Ragheb A., Ward S., Blecher G., Falcone M., Soave A., Dahlem R., van Renterghem K., Christopher N., Hatzichristodoulou G. (2022). ESSM Position Statement on Surgical Treatment of Peyronie’s Disease. Sex. Med..

[11] Lin H., Liu C., Wang R. (2017). Effect of Penile Traction and Vacuum Erectile Device for Peyronie’s Disease in an Animal Model. J. Sex. Med..

[12] Rosen R.C., Riley A., Wagner G., Osterloh I.H., Kirkpatrick J., Mishra A. (1997). The international index of erectile function (IIEF): A multidimensional scale for assessment of erectile dysfunction. Urology.

[13] Cappelleri J.C., Rosen R.C., Smith M.D., Mishra A., Osterloh I.H. (1999). Diagnostic evaluation of the erectile function domain of the International Index of Erectile Function. Urology.

[14] Shaw J.W., Reardon G., Sandor D.W., Rosen R.C., Ferguson D.M. (2010). Validation of stopwatch measurements of erection duration against responses to the sexual encounter profile and international index of erectile Function in patients treated with a phosphodiesterase type 5 inhibitor. J. Sex. Med..

[15] Hellstrom W.J.G., Feldman R., Rosen R.C., Smith T., Kaufman G., Tursi J. (2013). Bother and distress associated with peyronie’s disease: Validation of the Peyronie’s disease questionnaire. J. Urol..

[16] Neamatullah I., Douglass M.M., Lehman L.W., Reisner A., Villarroel M., Long W.J., Szolovits P., Moody G.B., Mark R.G., Clifford G.D. (2008). Automated de-identification of free-text medical records. BMC Med. Inform. Decis. Mak..

[17] Fernández-Pascual E., Manfredi C., Torremadé J., Ibarra F.P., Geli J.S., Romero-Otero J., García-Baquero R., Poblador A.F., Barbará M.R., Campos-Juanatey F. (2020). Multicenter Prospective Study of Grafting with Collagen Fleece TachoSil in Patients with Peyronie’s Disease. J. Sex. Med..

[18] Garaffa G., Trost L.W., Serefoglu E.C., Ralph D., Hellstrom W.J. (2013). Understanding the course of Peyronie’s disease. Int. J. Clin. Pract..

[19] Avant R.A., Ziegelmann M., Nehra A., Alom M., Kohler T., Trost L. (2019). Penile Traction Therapy and Vacuum Erection Devices in Peyronie’s Disease. Sex. Med. Rev..

[20] Yuan J., Lin H., Li P., Zhang R., Luo A., Berardinelli F., Dai Y., Wang R. (2010). Molecular mechanisms of vacuum therapy in penile rehabilitation: A novel animal study. Eur. Urol..

[21] Lue T.F., El-Sakka A.I. (1999). Lengthening shortened penis caused by Peyronie’s disease using circular venous grafting and daily stretching with a vacuum erection device. J. Urol..

